# Saphenous-sparing laparoscopic inguinal lymphadenectomy

**DOI:** 10.1590/S1677-5538.IBJU.2017.0120

**Published:** 2018

**Authors:** Gaetano Chiapparrone, Sebastiano Rapisarda, Bernardino de Concilio, Guglielmo Zeccolini, Michele Antoniutti, Antonio Celia

**Affiliations:** 1 Department of Urology, Ospedale do Cattinara, Trieste - Italy; 2 San Bassiano Hospital - Urology, Bassano del Grappa, Italy

## Abstract

**Introduction:**

Inguinal lymphadenectomy is an integral part in the management of penile cancer. Video endoscopic inguinal lymphadenectomy (VEIL) is emerging as a minimally invasive treatment to reduce postoperative complications.

**Materials and Methods:**

62 years old man underwent glansectomy for a squamous cell carcinoma (pT1b). At the physical examination one left inguinal lymph node was detectable (cN1). The chest-abdomen-pelvis CT was negative for metastasis. A 10-mm optical trocar and two 5mm operating trocar were placed. The optical trocar was placed in the apex of Scarpa's triangle after a skin incision and after the creation of a subcutaneous space by blunt finger dissection. The pCO2 was 8-10mmHg. The surgical technique involved the removal of superficial lymph nodes according to the scheme described by Deseler and of the deep lymph nodes. Sparing main venous structures and closing lymphatic vessels is important to reduce post operative complications. At the end of the procedure, a suction drain was placed per side.

**Results:**

Operative time was 90 minutes per side. Drains were removed on the seventh postoperative day. Hospital stay was 8 days and no postoperative complications occurred. The total number of nodes removed was 16 (8 per side) with 2 superficial positive nodes on the left side.

**Conclusion:**

ILND is burned by a high complication rate. VEIL provides a less invasive approach and a saphenous-sparing technique ensures a lower complication rate, reducing lymphorrhea, skin necrosis and wound complications (1–3). In experienced laparoscopic hands, VEIL is a safe and effective treatment.

## ARTICLE INFO

**Available at: http://www.intbrazjurol.com.br/video-section/20170120_Chiapparrone_et_al Int Braz J Urol. 2018; 44 (Video #8): 645-6**
